# An 8-bit monochrome palette of fluorescent nucleic acid sequences for DNA-based painting†

**DOI:** 10.1039/d2nr05269e

**Published:** 2022-11-18

**Authors:** Tadija Kekić, Jory Lietard

**Affiliations:** Institute of Inorganic Chemistry, University of Vienna Josef-Holaubek-Platz 2 1090 Vienna Austria jory.lietard@univie.ac.at

## Abstract

The ability to regulate, maintain and reproduce fluorogenic properties is a fundamental prerequisite of modern molecular diagnostics, nanotechnology and bioimaging. The sequence-dependence of the fluorescence properties in fluorophores commonly used in nucleic acid labelling is here being exploited to assemble a color scale in 256 shades of green Cy3 fluorescence. Using photolithography, we synthesize microarrays of labeled nucleic acids that can accurately reproduce 8-bit monochrome graphics by mapping color to fluorescence intensity and sequence. This DNA-based painting approach paves the way for a full RGB scale array fabrication process.

## Abstract 2

Using fluorescent labelling, graphical inputs in 8-bit monochrome format can be copied as DNA microarrays in 256 colours with high fidelity. This effort highlights the versatility of *in situ* microarray photolithography for surface patterning.

## Graphical abstract

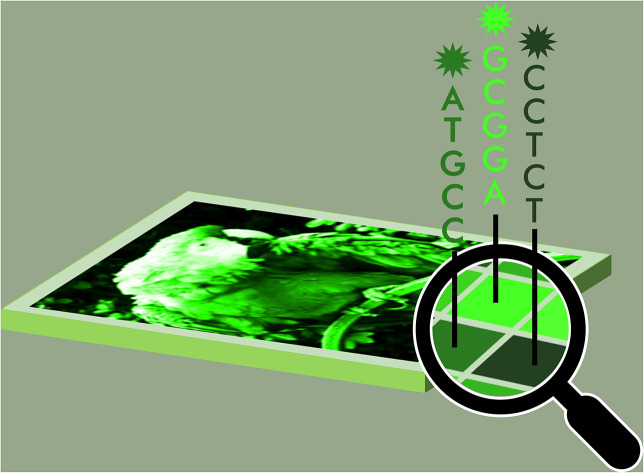

## Introduction

Access to libraries of nucleic acid sequences has helped the fields of genomics, synthetic biology, and DNA biotechnology to take a leap forward in scale and throughput.^1^ But pure numbers only partially unlock the potential of DNA nanotechnology. Indeed, having control over the distribution, identity and organization of all elements of the library is a fundamental aspect in the production of higher order nucleic acid structures,^2^ from synthetic genes^3–5^ to DNA origami^6^ but also in molecular circuitry,^7^ DNA computing,^8^ as well as next-generation sequencing.^9^ The spatial organization of nucleic acids as microarrays offers an additional level of sequence control as each surface-bound sequence is attributed a unique position. These two-dimensional arrangements can serve to reproduce the spatial distribution of RNA transcripts within a tissue sample in order to obtain valuable gene expression data at the single cell level.^10^ Nucleic acid microarrays can also be designed to generate informative molecular patterns for the purpose of authenticity or cryptography.^11,12^ All of these approaches rely on standard hybridization following Watson–Crick rules. In most cases, hybridization is detected by recording a fluorescent signal from a dye-labelled complementary strand. By allowing several dyes with appropriate excitation and emission properties to be used simultaneously, precise hybridization patterns on a microchip surface can be turned into a canvas where colour emerges from overlapping red, green and blue (RGB) channels.^13^ We recently showed that the sequence of an oligonucleotide serving as template for up to three simultaneous hybridization events can be fine-tuned in order to modulate hybridization affinity and in so doing, display any of eight shades of red, green and/or blue fluorescence, making it possible to assemble images in 256 colors.^14^ Achieving a greater range of DNA colorimetry in each RGB channel through hybridization alone is significantly more complex and requires a heavier use of mismatches and better control of temperature for duplex formation.

**Fig. 1:**
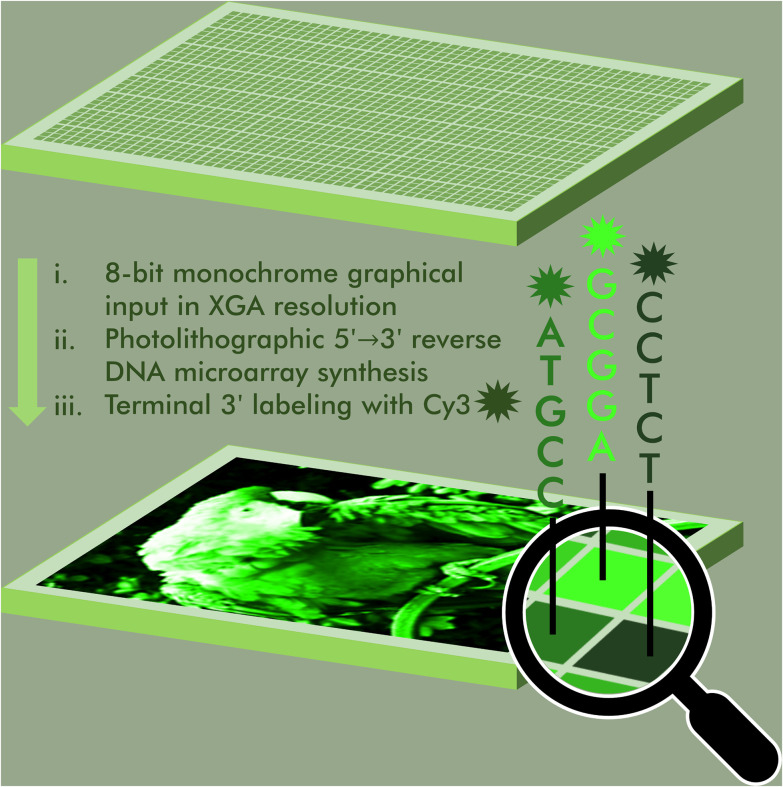
Schematic representation of the DNA painting process by microarray photolithography. A graphical input, in monochrome and of 1024 × 768 maximum resolution, is transformed into a Cy3-labeled DNA microarray. Colour intensity at the graphical level has a sequence equivalent and employs the sequence-dependent property of Cy3 fluorescence. DNA synthesis takes place using *in situ* microarray photolithography in the 5′ → 3′ direction, followed by terminal 3′-labeling with Cy3.

An alternative to hybridization for the construction of a colour gradient is the direct labelling of microarrays.^15^ Oligonucleotide arrays offers a unique approach to connect fluorescence intensity and sequence diversity. Indeed, the fluorescence of common cyanine and xanthene dyes is known to be sequence-dependent, with nucleobases in close proximity to the dye able to quench or enhance fluorescence through a mechanism involving π-stacking or electron transfer. Using photolithography, we have comprehensibly mapped the sequence-dependence of Cy3, Cy5, fluorescein (FAM) dyes in all 3′, 5′, single-stranded and double-stranded DNA formats.^16–20^ With cyanine dyes, we observe a positive influence of terminal guanines on fluorescence emission and a large quenching effect of terminal pyrimidines, especially cytosine. With fluorescein, the effect of purine and pyrimidines is reverse. While these observations largely hold true whether the dye is attached to the 5′ or the 3′ end, with sequences with a terminal dG being on average much brighter than any other base, we found sequence motifs in the 3′ series giving off intense fluorescence and which were absent from their 5′-labelled counterparts. These motifs contributed to the wider range of fluorescence intensity in 3′-Cy3 labelled DNA compared to 5′-Cy3. We exploited this range, wider than any other sequence-dependence study undertaken so far, to assemble a gradient of green colour. To extend this range even further, we introduced an additional parameter that controls the amount of UV light received during oligonucleotide synthesis, since photolysis marks the beginning of a new nucleoside coupling cycle in microarray photolithography. In so doing, we were able to calibrate a linear gradient of Cy3 fluorescence which was turned into an 8-bit colour scheme. Grayscale images up to 256 shades can be accurately reproduced as green scale microarray pictures purely from *in situ* labelled and *in situ* synthesized DNA and without the need for hybridization (Fig. 1).

## Results and discussion

### Exposure gradient and scale calibration

**Fig. 2:**
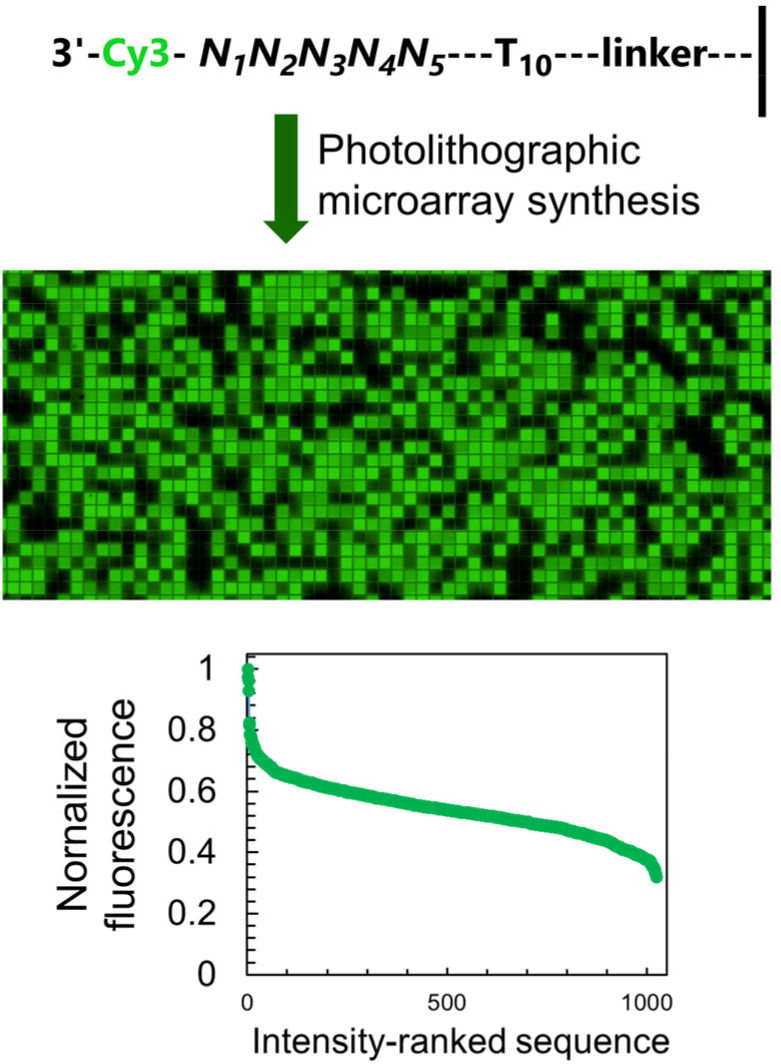
The fluorescence of Cy3 dye is sequence-dependent and is affected by the nature of the nucleotides immediately adjacent to the terminal label. The sequence-dependence was identified by permuting the nucleobases in a 5-nt long section at the 3′-end of single-stranded DNA oligonucleotides (1024 combinations) and by synthesizing all corresponding sequences *via* microarray photolithography followed by terminal labelling with a Cy3 phosphoramidite. A simple scan after synthesis reveals sequence-mediated effects on the fluorescence of Cy3, and a large range of intensity spanning a 65% difference between the brightest and darkest sequences.

To study how neighbouring nucleobases affect the fluorescence properties of common nucleic acid fluorophores, we synthesize microarrays containing all possible permutations of a 5-nt long stretch (1024 unique sequences, Fig. 2) immediately next to the terminal dye. The synthesis is carried out using UV photolithography and DNA phosphoramidites,^21–25^ either in the 3′ → 5′ or 5′ → 3′ direction^26^ and carrying an appropriate photoprotecting group (BzNPPOC^27^), followed by a final coupling of a Cy3/Cy5 phosphoramidite.

The sequence-dependent fluorescence of Cy3 and Cy5 when appended to the 3′ or 5′ end of single-stranded oligonucleotides was recently fully mapped and discussed in detail,^16,20^ with broad analogy between the two: guanine-rich sequences yielding bright fluorescence signals and C-rich analogs being significantly darker. Similarly, nucleotides further away from the terminus participate in affecting the fluorescence properties of the dye. It is however the span in signal intensity that differentiates the two datasets, with 3′-labelled single-stranded DNA displaying a larger range of intensities than for 5′ labelling (65% and 75% for Cy3 and Cy5, *versus* 50 and 65% with 5′ labeling^16^). This larger amplitude (Table S1, ESI†) prompted us to envisage the creation of a colour scale gradient from fluorescently-tagged oligonucleotide arrays at the 3′-end. We chose to focus on Cy3 because of the well-known tendency of Cy5 to photobleach under repeating exposure to 635 nm excitation^28,29^ and we started with the complete set of 4^5^ permutations. Not only are the positions 4 and 5 nt away from the terminus relevant for fluorescence modulation,^20^ but a permutation set of only 3 nucleotides would be insufficient to yield an 8-bit range (4^3^, 64 permutations).

**Fig. 3:**
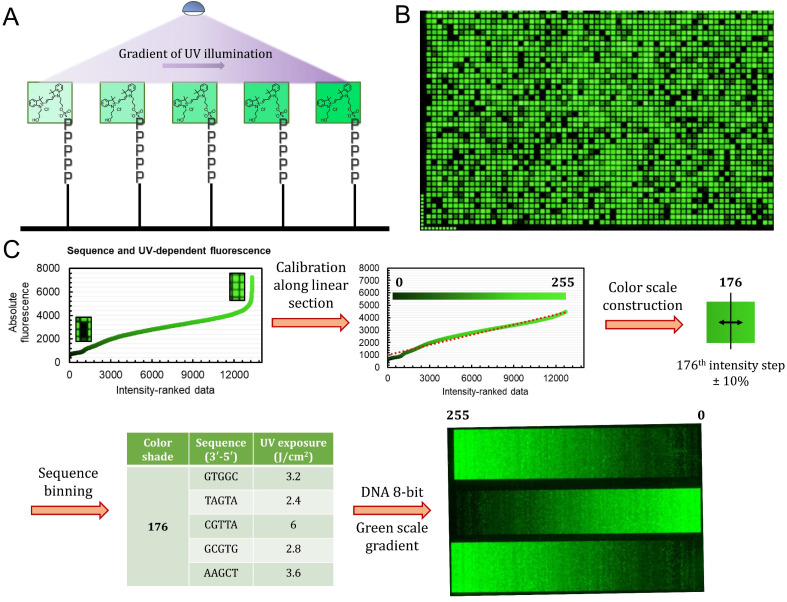
Process of implementing a gradient of UV exposure to generate a full 8-bit gradient of colour. (A) Schematic illustration of Cy3 incorporation efficiency as a function of UV exposure immediately prior to the coupling of the fluorophore phosphoramidite. (B) Excerpt of a microarray scan (∼15% of the total synthesis area) of the sequence-dependent fluorescence with each 5mer exposed to each of the 13 increments of UV light. (C) The calibration process started by ranking all 13 312 datapoints in order of increasing fluorescence. Extreme values (>4500 a.u.) were discarded in order to obtain a linearly increasing intensity plot. The intensity range was then divided into 256 shades of green and a margin of ±10% error was introduced in order to create bins of sequence/exposure pairs (5 elements per bin). A 256-color gradient (50 × 256 pixels) was then synthesized as a DNA microarray by first generating an 8-bit grayscale input bitmap of 1024 × 768 resolution. Using a custom python script, each time a specific shade (0–255) is called, one sequence/exposure pair from the equivalent bin is randomly allocated to the corresponding pixel coordinate. The DNA microarray is then synthesized and deprotected according to published procedures.

The sequence-dependence alone is however not enough to construct a linear gradient reaching down to background noise, despite having access to 1024 datapoints. Indeed, since the signal intensities of permutation sequences have normal distribution; when sorted by value, the distribution of intensities adopt a sigmoidal shape (Fig. 2). The central area of the sigmoidal is approximately linear and sequences belonging to this area can be used in grayscale microarray fabrication. However, the intensity range is fairly narrow and significantly above background fluorescence. In order to create a larger range of linearly increasing fluorescence signals reaching down to background, we introduced a gradient of UV illumination at the penultimate step of microarray synthesis just before incorporation of the dye. This gradient creates a range of photodeprotection efficiency of the 3′-BzNPPOC group that affects the total amount of dye that can be coupled to the 3′ end of oligonucleotides (Fig. 3A).

The UV exposure of the final 3′-BzNPPOC group was conducted in increments of 0.4 J cm^−2^, from 0 (no photolysis) to 4 J cm^−2^ (∼95% photolysis efficiency) and with additional steps at 5 and 6 J cm^−2^. Smaller increments would result in finer control over the photolysis rate and increase the density of fluorescent data points, but are in practice difficult to implement due to communication between software controller, electronic shutter and DMD (Digital Micromirror Device). Each of the 1024 sequence permutations was therefore subjected to 13 different exposure steps, creating an ensemble of >13 000 unique points (Fig. 3B). The range of fluorescence, while still adopting a normal distribution, now reaches very low brightness levels, particularly for poorly exposed C-rich sequences. This extended fluorescent space allows for a colour scale to be constructed in a linear fashion, starting from barely exposed and unexposed states to bright, fully exposed, fluorescence-enhancing sequences. To do so, we first trimmed the ranked data (Fig. 3C) to exclude extreme brightness points (>4500 a.u.) as they would not allow for a direct dark → bright line to be drawn. The now linear section of the fluorescence distribution (*r*^2^ = 0.974) still contains >12 000 data points, which is sufficient to establish an 8-bit gradient of green colour. After calibration, the brightness range was divided into 256 steps of equal fluorescence increase and to each step was attributed the corresponding sequence and its exposure parameter. We also included data points that deviate up to ±10% from the selected intensity position so as to create a pool of suitable elements allocated to each colour shade. This is relevant in order to negate variability in synthesis efficiency between microarrays.

With all 256 bins populated with 5 sequence/exposure pairs, we then created a script that transforms any 8-bit grayscale input image into a microarray synthesis design by allocating a random sequence/exposure pair from the bin of corresponding intensity to each pixel of the input (ESI S1†). The resulting output is therefore of the same resolution and same intensity per pixel coordinate. We synthesized a series of 8-bit colour ramps and scanning at 532 nm reveals correct and reproducible 256-color green scale painting purely from *in situ*-labelled DNA oligonucleotides (Fig. 3C). The darkest shades correspond to background fluorescence and there is smooth transitioning in green intensity.

The process is however not error-free as we note the presence of unexpected brighter lines in the darker region of the spectrum (0–25). On average, these artefacts correspond to sequences exposed to a small fraction of the total UV. Given that the photolysis rate of NPPOC and derivatives is exponential at low exposure doses (0–2 J cm^−2^), minute differences in actual UV irradiance could cause significant shifts in photolysis efficiency. The sources of exposure errors could come from temporary fluctuations in the power output of the LED, or more likely from unintended exposure of neighbouring features, which is particularly relevant here given the minimal distance between individual pixels (∼1 μm).^21^ The use of larger feature sizes should reduce the impact of unintended photodeprotection since smaller features are also more susceptible to receiving stray light.^21,25,30^

### Green scale DNA printing

**Fig. 4:**
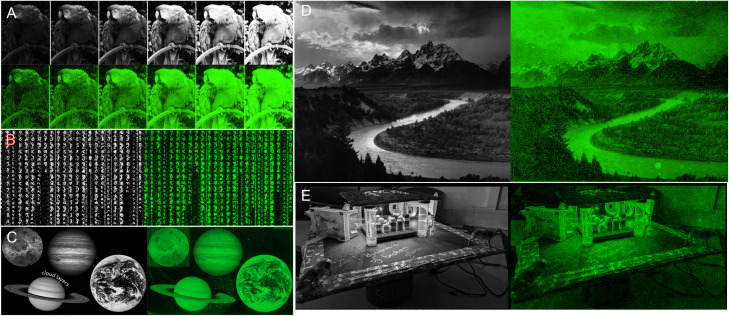
DNA painting with fluorescently-labelled single-stranded oligonucleotides for the reproduction of digital 8-bit grayscale images (A–E). In grayscale are the input digital files (of 1024 × 768 maximum resolution) and in green high-resolution scans (2.5 μm resolution) of the Cy3-labeled DNA arrays after bin allocating, synthesis and deprotection. The parrot input A is a series of contrast adjustments. All images are CC-BY except for image E which comes from Dr Lietard's personal records.

We next applied our colour gradient in more complex patterns by attempting to reproduce 8-bit grayscale digital images in fluorescent DNA array format. After downscaling and grayscale conversion, the input images were transformed into synthesis instructions and the resulting fluorescent microarrays were scanned at 532 nm following synthesis and DNA deprotection. Scans, together with their original grayscale input, are shown in Fig. 4. DNA facsimiles reproduce a digital input with a fair amount of accuracy, displaying sufficient contrast to distinguish all major elements with the exception of Jupiter's cloud layers (Fig. 4C). The study on contrast adjustment in the parrot sample (Fig. 4A) works well at high contrast with a clear increase in colour saturation. The painting process is however only moderately able to work at lower contrast settings, where the outline of the animal's body remains somewhat visible despite the corresponding input being uniformly dark. This could again be due to variations in UV power and to assigning low exposure doses (0.4 J) to pixel intensity >0. Extreme saturation does not transform into burnt-out white colour since the gradient of green does not extend to white. Pixel saturation during scanning would be possible, for instance by adjusting the photomultiplier gain of the laser, but at the risk of increased noise. Some noise is noticeable already in the fluorescent images (Fig. 4D), particularly in the darker areas, giving them a semblance of dithering. While some of it may be due to locally higher photolysis efficiency, the perceived graininess of the images is perhaps a direct effect of micromirror placement in the DMD with a 1 μm distance between each mirror. This inaccessible space between pixels effectively creates a grid which isolates features from one another and giving an impression of pixel discontinuity. Larger feature sizes will reduce the impact of the unexposed grid, but at the cost of total image size.

**Fig. 5:**
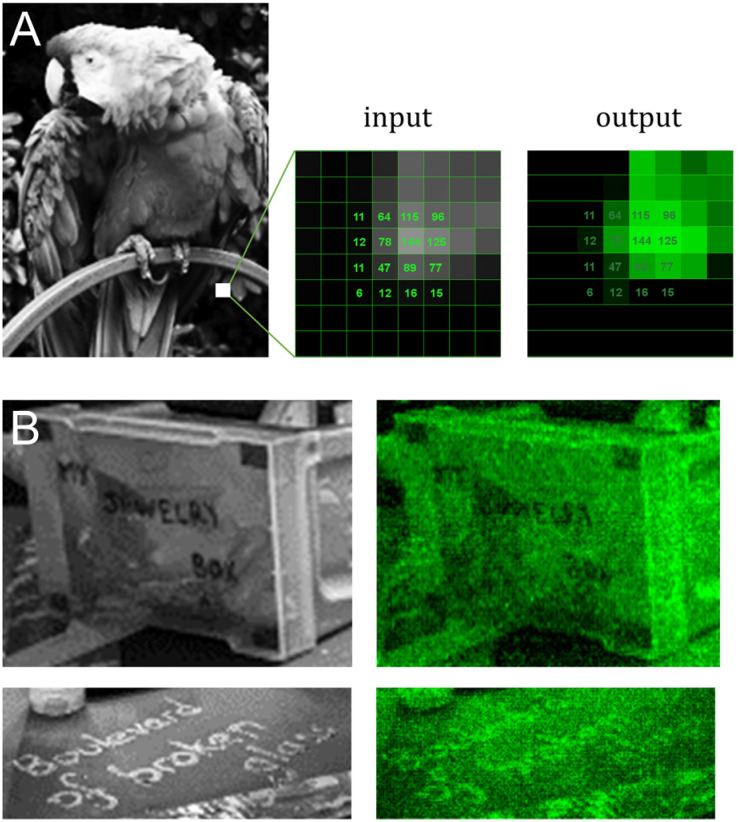
Detail analysis of input/output images from Fig. 4A and E. (A) Cropped-out section (8 × 8 pixels) of the 8-bit input and output parrot sample with the corresponding pixel shade intensity in a 4 × 4 central grid. The output image reproduces the grayscale canvas with good accuracy with the exception of color value 12, 77 and 125 appearing too bright relative to adjacent pixels. (B) Cropped-out and zoomed-in section of the mortarboard displaying various inscriptions. The message in block letters is legible, but fluorescent cursive writing requires the input to be fully decipherable.

Messages are distinguishable, despite imperfections, even in regions with low contrast between letters and background (Fig. 4E and Fig. 5B). To estimate the amount of error in actual colour value in the fluorescent output, we selected a small area of the input bird image and assigned the corresponding pixel intensity in 4 × 4 grid (Fig. 5A). The fluorescent output image has at least three colour values that are not accurately converted (12, 77 and 125), adding up to 4 pixels out of 16 that are visibly off, or a ∼25% error-rate. This error-rate should be understood as apparent deviation from the intended value and is the most likely source of granularity in the fluorescent images. Further refinement of bin and intensity allocation should be possible through an iterative process of sorting out sequence/exposure pairs that do not perform properly, but a single pair of sequence/exposure per shade also removes the ability to correct for errors in synthesis, *i.e.* in the case of poor coupling of a given nucleoside phosphoramidite. Another approach to reduce the error-rate would be to start with a larger set of pentamers that would include non-canonical nucleobases. Not only would they enrich the list of sequences that can be picked out for each intensity shade, they could also potentially increase the range of Cy3 fluorescence intensity beyond what is currently achievable with A, C, G and T.

## Conclusions

In this study, we showed how the spatial organization of DNA strands as microarrays can be used to convey pictorial information without any need for hybridization. We used the inherent ability of nucleic acid sequences to modulate the fluorescence properties of conjugated dyes to create a colour scale containing 256 shades (8-bit colour coding). To do so, we harnessed the sequence-dependence of Cy3 fluorescence in terminally-labelled single stranded DNA, and especially the largest available intensity span when Cy3 is attached at the 3′ end, to start the process of assigning colour/fluorescence intensity to a specific pentamer. Combining the 3′-Cy3 sequence-dependence with Cy3 coupling efficiency using a gradient a UV exposure allowed for the range of fluorescence intensities to be expanded further and to yield very low signals, down to background level. The corresponding dataset was then used to calibrate a linear gradient of increasing light intensity, making it possible to print fluorescent, DNA-based reproduction of 8-bit grayscale digital images. Synthesis and DNA deprotection are sufficient to reveal the molecular pictures and the process is here shown for green fluorescence, but should be applicable to red and blue channels as well using Cy5 and FAM, respectively. Indeed, the fluorescence of Cy5 and FAM was also found to be sequence-dependent and nucleotide patterns influencing the intensity of these dyes have been previously identified.^19,20^ Conceptually, the synthesis of DNA microarrays including all three RGB dyes would require the preparation of three individual DNA strands per feature, each being selectively terminable with Cy3, Cy5 or FAM. A three-pronged branching unit would serve as the ideal starting point for the synthesis of three branches each terminated with a unique dye. As such, the work described herein represents the first step towards an 8-bit RGB system for DNA-based painting.

## Author contributions

T. K. performed all experiments and analyzed the data while J. L. provided supervision, designed the experiments, acquired funding and wrote this manuscript, which all authors read and approved.

## Conflicts of interest

There are no conflicts to declare.

## Supplementary Material

NR-014-D2NR05269E-s001

## References

[cit1] Song L. F., Deng Z. H., Gong Z. Y., Li L. L., Li B. Z. (2021). Front. Bioeng. Biotechnol..

[cit2] Gu R. , Tang L., Aritome I. and Zauscher S., in Polymer and Biopolymer Brushes, 2017, pp. 627–654

[cit3] Menon G., Krishnan J. (2019). ACS Synth. Biol..

[cit4] Sidore A. M., Plesa C., Samson J. A., Lubock N. B., Kosuri S. (2020). Nucleic Acids Res..

[cit5] Kosuri S., Church G. M. (2014). Nat. Methods.

[cit6] Schmidt T. L., Beliveau B. J., Uca Y. O., Theilmann M., Da Cruz F., Wu C. T., Shih W. M. (2015). Nat. Commun..

[cit7] Chirieleison S. M., Allen P. B., Simpson Z. B., Ellington A. D., Chen X. (2013). Nat. Chem..

[cit8] Chatterjee G., Dalchau N., Muscat R. A., Phillips A., Seelig G. (2017). Nat. Nanotechnol..

[cit9] Goodwin S., McPherson J. D., McCombie W. R. (2016). Nat. Rev. Genet..

[cit10] Stahl P. L., Salmen F., Vickovic S., Lundmark A., Navarro J. F., Magnusson J., Giacomello S., Asp M., Westholm J. O., Huss M., Mollbrink A., Linnarsson S., Codeluppi S., Borg A., Ponten F., Costea P. I., Sahlen P., Mulder J., Bergmann O., Lundeberg J., Frisen J. (2016). Science.

[cit11] Holden M. T., Smith L. M. (2019). ACS Comb. Sci..

[cit12] Schaudy E., Somoza M. M., Lietard J. (2020). Chem. – Eur. J..

[cit13] Pardatscher G., Schwarz-Schilling M., Daube S. S., Bar-Ziv R. H., Simmel F. C. (2018). Angew. Chem., Int. Ed..

[cit14] Holz K., Schaudy E., Lietard J., Somoza M. M. (2019). Nat. Commun..

[cit15] Carbonell C., Valles D., Wong A. M., Carlini A. S., Touve M. A., Korpanty J., Gianneschi N. C., Braunschweig A. B. (2020). Nat. Commun..

[cit16] Agbavwe C., Somoza M. M. (2011). PLoS One.

[cit17] Kretschy N., Somoza M. M. (2014). PLoS One.

[cit18] Kretschy N., Sack M., Somoza M. M. (2016). Bioconjugate Chem..

[cit19] Lietard J., Ameur D., Somoza M. M. (2022). RSC Adv..

[cit20] Kekić T., Lietard J. (2022). Sci. Rep..

[cit21] Agbavwe C., Kim C., Hong D., Heinrich K., Wang T., Somoza M. M. (2011). J. Nanobiotechnol..

[cit22] Sack M., Holz K., Holik A. K., Kretschy N., Somoza V., Stengele K. P., Somoza M. M. (2016). J. Nanobiotechnol..

[cit23] Holz K., Lietard J., Somoza M. M. (2017). ACS Sustainable Chem. Eng..

[cit24] Lietard J. , Damha M. J. and Somoza M. M., in Enzymatic and Chemical Synthesis of Nucleic Acid Derivatives, ed. J. Fernández-Lucas, 2018

[cit25] Lietard J., Leger A., Erlich Y., Sadowski N., Timp W., Somoza M. M. (2021). Nucleic Acids Res..

[cit26] Holz K., Hoi J. K., Schaudy E., Somoza V., Lietard J., Somoza M. M. (2018). Sci. Rep..

[cit27] Kretschy N., Holik A. K., Somoza V., Stengele K. P., Somoza M. M. (2015). Angew. Chem., Int. Ed..

[cit28] Staal Y. C. M., van Herwijnen M. H. M., van Schooten F. J., van Delft J. H. M. (2005). BMC Genomics.

[cit29] von der Haar M., Heuer C., Pahler M., von der Haar K., Lindner P., Scheper T., Stahl F. (2016). Biology.

[cit30] Garland P. B., Serafinowski P. J. (2002). Nucleic Acids Res..

