# Supplementary material for: An 8-bit monochrome palette of fluorescent nucleic acid sequences for DNA-based painting
Source: Nanoscale. 2022 Nov 18;14(47):17528–33. doi: 10.1039/d2nr05269e (PMC9730302; doi:10.1039/d2nr05269e)
Supplement: NR-014-D2NR05269E-s001 [file NR-014-D2NR05269E-s001.pdf]

Electronic Supplementary Material (ESI) for Nanoscale.
This journal is © The Royal Society of Chemistry 2022

Supplementary Information

An 8-bit monochrome palette of fluorescent nucleic acid sequences for DNA-based painting

Tadija Kekić and Jory Lietard*

Institute of Inorganic Chemistry, Faculty of Chemistry, University of Vienna, Vienna, Austria

*jory.lietard@univie.ac.at

Table of Contents

Experimental Procedures 2
  Sequence Design 2
  Microarray Synthesis 2
  Data Extraction and Analysis 3
  UV exposure gradient and scale calibration 3
  Green scale DNA printing 3
Additional Data 4
References 15

Experimental Procedures

Sequence Design

The design of DNA oligonucleotides is based on a permutation scheme allowing for the parallel synthesis of all possible combinations of 5 consecutive nucleotides immediately adjacent to a terminal fluorescent dye. The total amount of combinations is 4^5, or 1024 unique sequences. Downstream of the permutation sequence (P_1 to P_5), an adjustment section of 15 nucleotides is added, separated from the permutation section with a T_{15} spacer. The adjustment section can be represented in the form N_1 to N_5, where each N_x is a trinucleotide in the form "ACGT – P_x". Therefore, if P_1 is T, N_1 is ACG. This adjustment section serves as a way to guarantee the same amount of A, C, G and T nucleotides in each oligonucleotide, regardless of the nature of the permutation. This is important because of the slightly lower coupling efficiency of G phosphoramidites, which would result in a lower amount of G-rich sequences and a deceptively lower fluorescence. By keeping a constant nucleotide content, the synthesis efficiency is expected to be largely homogenous across all 1024 oligonucleotides. A final T_5 linker is added to further distance the oligonucleotide sequence from the microarray surface. Conceptually, the oligonucleotide design can be represented as follows:

$$Dye — P_1P_2P_3P_4P_5 — T_{15} — N_1N_2N_3N_4N_5 — T_5 — glass \text{ with } N_x = (ACGT - P_x)$$

Microarray Synthesis

The synthesis of oligonucleotide by Maskless Array Synthesis (MAS) follows previously published procedures that have seen several rounds of technical and experimental improvements over time[1-7]. The synthesis occurs on glass microscope slides (Schott Nexterion D) which have been silanized with hydroxybutyramide prior to synthesis. Synthesis follows standard phosphoramidite chemistry with 3'-protected 5' nucleoside phosphoramidites and is therefore of 5'→3' directionality (so-called "reverse mode"). The sole difference with conventional solid-phase synthesis is the use of a photosensitive protecting group (benzyl-NPPOC, bzNPPOC) instead of the acid-sensitive DMTr group[8]. Photodeprotection is accomplished by exposing the BzNPPOC to 365 nm UV light while the microarray slide is immersed in 1% solution of imidazole in DMSO. UV light, generated by a UV-LED (Nichia), is reflected by a Digital Micromirror Device (DMD, Texas Instruments) as a pattern corresponding to "ON" and "OFF" mirrors, where light reflected from "ON" mirrors will be imaged onto the array surface, triggering photodeprotection on the corresponding area. "OFF" mirrors reflect UV light away from the surface, where the corresponding area will remain unexposed. Only exposed areas and the correspondingly 5'-OH photodeprotected oligonucleotides will be able to couple with the next incoming phosphoramidite (3'-BzNPPOC, Orgentis), while unexposed areas will remain unaffected by the coupling event. Phosphoramidites are prepared as 30 mM solutions in dry ACN and coupled for 60 s in 0.25 M dicyanoimidatole in ACN. After coupling, unreacted 5'-OH are capped by performing a second coupling event with a DMTr-dT phosphoramidite which essentially counts as capping since DMTr group are not removed in MAS. The final coupling is the labelling with Cy3 phosphoramidite

(LinkTech), which is performed with a 50 mM solution of Cy3 in ACN and with 2 x 5 min coupling time. Photolysis is carried out by exposing the selected area to a radiant exposure of 6 J/cm^2 (~1 min at 100 mW/cm^2 irradiance) which removes 99% of BzNPPOC groups. The delivery of reagents and solvents to the microarray surface encased in a custom-made reaction chamber is done with an automated DNA synthesizer (Expedite 8909, PerSeptive Biosystems).

Data Extraction and Analysis

After synthesis, the microarrays are immediately washed in ACN for 2 h at r.t. to remove unbound Cy3, dried in a microarray centrifuge then scanned on a microarray scanner at 5 or 2.5 µm resolution (GenePix 4100A or 4400A) using a 532 nm laser excitation wavelength. After the initial scanning, the arrays are transferred in a 1:1 solution of ethylenediamine in ethanol in a glass jar overnight at r.t. to remove phosphate and base protecting groups. The arrays are then washed in 2 × 20 ml H_2O, dried, and scanned again. Scan images in .tif format are analysed with NimbleScan (NimbleGen) and the extracted data processed with Excel. The reported fluorescence intensity data are an average across all replicates, are corrected for background fluorescence and are the result of at least three independent measurements. Background fluorescence corresponds to the fluorescence intensity of a non-labelled version of each sequence and each exposure parameter.

UV exposure gradient and scale calibration

The gradient of UV illumination performed immediately prior to dye coupling ranged from 0 to 6 J/cm^2 of total irradiance, where ~ 4J/cm^2 is expected to remove ~95% of 3′-BzNPPOC protecting groups. UV illumination was carried out in incremental steps of 0.4 J from 0 to 4 J/cm^2, along with two additional exposure steps at 5 and 6 J/cm^2, where the photolysis rate has reached a plateau. Smaller increments are difficult to reliably measure because of incompressible time for electronic communication between DMD and shutter. With a total of 13 exposure increments for each of the 1024 sequence permutations, a total of 13312 elements was generated. The synthesis of the exposure gradient for the 3′ terminally-labeled sequence permutation library was otherwise carried out as explained in the previous section. After synthesis and deprotection, the overall range of fluorescence was ranked by intensity, there again adopting a sigmoidal shape whose darkest point corresponds to background fluorescence, i.e. sequences exposed for 0 J/cm^2 (no labeling). The linear section of the curve was selected to construct a linear gradient of fluorescence brightness and signal intensity was divided into 256 increments, or bins. To account for potential errors in identifying a single suitable sequence/exposure tandem for each bin, we instead brought together all elements within ±10% of each incremental fluorescence and randomly chose 5 elements to be representative of each of the 256 bins.

Green scale DNA printing

Input grayscale images in 8-bit format, from public domain or from personal records, were first downscaled to a maximum of 1024 × 768 resolution. The intensity value for each pixel as well as its Cartesian coordinate was fed into a python script that randomly allocates a sequence and its exposure

rate from the bin of corresponding signal intensity. The result grid input was then transformed into a series of digital masks and synthesis instructions using MatLab (MathWorks) and a script designed in-house.

Additional Data

Supplementary File S1. Python script for the automated attribution of a sequence/exposure pair to any given grayscale color intensity in 8-bit monochrome graphical input.

```
from PIL import Image
import numpy as np
import random

# TAKES SINGLE LINED TXT FILE OF SEQUENCES WITH NAMES (RANKED FROM LOWEST TO HIGHEST) AND MAKES AN ARRAY OUT OF IT
RealSeqRange = np.loadtxt("SelectedSeqGradRange.txt", dtype=str)
RealSeqRange = RealSeqRange.reshape((len(RealSeqRange), 1), order='F')
# print(RealSeqRange)

RealSeqIndex = np.loadtxt("SelectedSeqGradIndex.txt", dtype=str)
RealSeqIndex = RealSeqIndex.reshape((len(RealSeqIndex), 1), order='F')
# print(RealSeqIndex)

RealSeqCount = np.loadtxt("SelectedSeqGradCount.txt", dtype=str)
RealSeqCount = RealSeqCount.reshape((len(RealSeqCount), 1), order='F')
# print(RealSeqCount)
SequenceListFinal = [None] * (256*5)  # from 256
# print(len(RealSeqCount))
# print(len(RealSeqRange))
random.seed(1)
countA = 0
countB = 0
CountC = 0
```

```
for i in range(256):
    countB = countB + int(str(RealSeqCount[i])[2:-2])
    # print(countA, countB)
    y1 = random.choice(range(countA, countB))
    y2 = random.choice(range(countA, countB))
    y3 = random.choice(range(countA, countB))
    y4 = random.choice(range(countA, countB))
    y5 = random.choice(range(countA, countB))
    countA = countB + 1
    # print(countA)
    SequenceListFinal[CountC] = str(RealSeqRange[y1])[2:-3]
    CountC = CountC + 1
    SequenceListFinal[CountC] = str(RealSeqRange[y2])[2:-3]
    CountC = CountC + 1
    SequenceListFinal[CountC] = str(RealSeqRange[y3])[2:-3]
    CountC = CountC + 1
    SequenceListFinal[CountC] = str(RealSeqRange[y4])[2:-3]
    CountC = CountC + 1
    SequenceListFinal[CountC] = str(RealSeqRange[y5])[2:-3]
    CountC = CountC + 1

with open('BinnedSeq8bit.txt', 'w') as file_handle:
    for list_item in SequenceListFinal:
        file_handle.write('%s\n' % list_item)
# OPENS INPUT BMP FILE AS ARRAY
Img = Image.open('ExperimentTest3.bmp')  # from ExperimentTest3
Img = np.array(Img)

# CONTROL
# print(Img)

# MODIFIES INPUT ARRAY IN A SINGLE LINE FILE GOING FROM 0-768 (for 1024 times)
grid = (Img.reshape((786432, 1), order='F'))
# print(grid)

# SAVES THE GRID FILE AS TXT AND OPENS IT AS A LIST
np.savetxt("single_line.txt", grid, delimiter="", fmt='%5.0f')

Sequence = np.array(grid)
Sequence = Sequence.astype('str')
```

```
SequenceList = Sequence.tolist()

# OLD PART THAT DEFINES BACKGROUND IN COLOURED BMP INPUT
# for i in range(len(SequenceList)):
#     if SequenceList[i]==['7']:
#         SequenceList[i] = 's,Background'

# TAKES SINGLE LINED TXT FILE OF SEQUENCES WITH NAMES (RANKED FROM LOWEST TO HIGHEST) AND MAKES AN ARRAY OUT OF IT
RealSeq = np.loadtxt("BinnedSeq8bit.txt", dtype=str)
RealSeq = RealSeq.reshape((len(RealSeq), 1), order='F')
# print(RealSeq)

# SEED IS NOT NEEDED BUT STAYS FOR TESTING JUST IN CASE
random.seed(1)

# LOOPS OVER ARRAY TO MATCH GRAYSCALE PIXEL VALUE-LOCATION TO ALLOCATED SEQUENCE (per SEQ MAP)
# AND EXPORT A FILE READY FOR INPUT IN MATLAB PROGRAM
SequenceListFinal = SequenceList
x = (RealSeq.tolist())

# len(SequenceList)
j = 0
for i in SequenceList:
    y = random.choice(range((int(str(i)[2:-2])*5), ((int(str(i)[2:-2])*5)+5)))
    SequenceListFinal[j] = str(x[y])[3:-2]
    j = j + 1

with open('SeqForMatlab8bit.txt', 'w') as file_handle:
    for list_item in SequenceListFinal:
        file_handle.write('%s\n' % list_item)

# CORRECTES FOR EXPOSURE PRIOR TO CY3 COUPLING

ExposureSeq1 = np.loadtxt("SeqForMatlab8bit.txt", dtype=str)
ExposureSeq1 = ExposureSeq1.reshape((len(ExposureSeq1), 1), order='F')
ExposureSeq1 = ExposureSeq1.tolist()

ExposureSeq2 = np.loadtxt("SeqForMatlab8bit.txt", dtype=str)
```

```
ExposureSeq2 = ExposureSeq2.reshape((len(ExposureSeq2), 1), order='F')
ExposureSeq2 = ExposureSeq2.tolist()

for i in range(len(ExposureSeq1)):
    ExposureSeq1[i] = str(ExposureSeq1[i])[-5:-2]
    ExposureSeq2[i] = str(ExposureSeq2[i])[37:-2]
    if str(ExposureSeq1[i]) == str(0.4):
        ExposureSeq2[i] = str("9" + str(ExposureSeq2[i]))
    if str(ExposureSeq1[i]) == "0.8":
        ExposureSeq2[i] = str("99" + str(ExposureSeq2[i]))
    if str(ExposureSeq1[i]) == "1.2":
        ExposureSeq2[i] = str("999" + str(ExposureSeq2[i]))
    if str(ExposureSeq1[i]) == "1.6":
        ExposureSeq2[i] = str("9999" + str(ExposureSeq2[i]))
    if str(ExposureSeq1[i]) == "2.0":
        ExposureSeq2[i] = str("99999" + str(ExposureSeq2[i]))
    if str(ExposureSeq1[i]) == "2.4":
        ExposureSeq2[i] = str("999999" + str(ExposureSeq2[i]))
    if str(ExposureSeq1[i]) == "2.8":
        ExposureSeq2[i] = str("9999999" + str(ExposureSeq2[i]))
    if str(ExposureSeq1[i]) == "3.2":
        ExposureSeq2[i] = str("99999999" + str(ExposureSeq2[i]))
    if str(ExposureSeq1[i]) == "3.6":
        ExposureSeq2[i] = str("999999999" + str(ExposureSeq2[i]))
    if str(ExposureSeq1[i]) == "4.0":
        ExposureSeq2[i] = str("9999999999" + str(ExposureSeq2[i]))
    if str(ExposureSeq1[i]) == "5.0":
        ExposureSeq2[i] = str("99999999999" + str(ExposureSeq2[i]))
    if str(ExposureSeq1[i]) == "6.0":
        ExposureSeq2[i] = str("999999999999" + str(ExposureSeq2[i]))

with open('SeqForMatlabExposure8bit.txt', 'w') as file_handle:
    for list_item in ExposureSeq2:
        file_handle.write('%s\n' % list_item)
```

Table S1. Fluorescence rankings of 3′-Cy3 labeled ssDNA of all possible pentamers 5′ to the dye. Fluorescence is normalized to that of the brightest sequence combination.

Sequence:	Norm. fluor.	*SEM*
GCGCG	1.000	0.035
GCGCT	0.964	0.064
GCGCC	0.944	0.034
GCGCA	0.876	0.027
GGCGC	0.793	0.047
AGCGC	0.792	0.034
AGGGG	0.757	0.038
AGGGT	0.725	0.054
AGGGC	0.715	0.052
AGGCC	0.714	0.045
TGCGC	0.697	0.048
AGGCG	0.689	0.033
AGGAG	0.669	0.059
CGCGC	0.660	0.052
ACGCG	0.657	0.043
GGGCC	0.655	0.076
GGGGC	0.650	0.045
AGGCT	0.648	0.024
AGGGA	0.642	0.037
AGGTT	0.638	0.054
GGGGG	0.635	0.030
AGGAA	0.635	0.045
TGGGG	0.626	0.053
TGGCC	0.624	0.064
AGCGG	0.618	0.069
AGGAT	0.617	0.032
AGCGT	0.616	0.044
TGGGC	0.616	0.068
GGGGT	0.613	0.042
GCGGC	0.611	0.053
AGGTG	0.606	0.037
AGGTC	0.605	0.047
GGCGG	0.604	0.054
AGGAC	0.600	0.034
AGGTA	0.599	0.041
AAGGG	0.596	0.068
AGAGG	0.594	0.040
AGAGT	0.593	0.044
TGGTT	0.593	0.047
GGGGA	0.591	0.028
AGAAT	0.589	0.024
AGGCA	0.588	0.029
TGGGA	0.587	0.027
TGGGT	0.586	0.044
GGGTG	0.586	0.041
TGGAA	0.585	0.052
GGCAG	0.585	0.076
AGAAG	0.582	0.029
AGCGA	0.580	0.052
AGTAT	0.579	0.047
AGTAC	0.579	0.038
GGGCA	0.579	0.050
GGCGA	0.577	0.042
GGGAC	0.574	0.026
AGAAA	0.568	0.030
TGGCG	0.566	0.036
TGGCA	0.565	0.045
GGCCG	0.564	0.072
AGTGT	0.564	0.039
GGGTC	0.563	0.043
GGGCG	0.563	0.045
GGCCC	0.562	0.072
TGGTA	0.561	0.028
AGTCT	0.560	0.037
TGGAG	0.560	0.050
AAGGA	0.559	0.021
GGGCT	0.558	0.044
GGGAA	0.558	0.029
TGCGG	0.558	0.057
TGGTC	0.557	0.035
AAGGT	0.557	0.029
AGTGG	0.555	0.040
AGTTA	0.555	0.030
AGCCC	0.554	0.034
GGGAG	0.553	0.041
GGCAA	0.552	0.040
AGACG	0.552	0.035
AGCCT	0.552	0.031
GGCCA	0.551	0.059
CGCGG	0.551	0.064
GGGAT	0.550	0.037
GGGTT	0.549	0.029
GGGTA	0.548	0.037
AGCTG	0.548	0.034
AGTTG	0.548	0.046
AAGGC	0.548	0.058
AGCCG	0.547	0.034
GGTGA	0.546	0.076
TGGTG	0.544	0.031
AGAAC	0.543	0.026
TGGAT	0.543	0.033
GGCGT	0.543	0.032
GGCCT	0.539	0.037
TGAGT	0.539	0.029
TGAGG	0.537	0.048
AGTGA	0.536	0.037
TGGAC	0.534	0.037
GGAGG	0.534	0.029
GGAGC	0.534	0.034
AAAGG	0.534	0.035
AGCTT	0.533	0.067
AGATG	0.533	0.039
AAGAT	0.533	0.052
CGGGG	0.532	0.033
AGATT	0.531	0.036
AGTGC	0.531	0.037
AGACT	0.531	0.036
AGTTC	0.530	0.070
AGAGC	0.530	0.038
TGAAG	0.529	0.056
GGAGT	0.529	0.043
AGATC	0.529	0.026
AAGTT	0.527	0.030
AGACC	0.527	0.028
GCGAG	0.526	0.059
AAGCG	0.525	0.028
GGCAC	0.525	0.031
AGTCG	0.525	0.036
GGAGA	0.525	0.055
AGTTT	0.524	0.030
AAGCT	0.524	0.043
AGTAA	0.524	0.030
CGCGA	0.523	0.038
AAAGT	0.522	0.041
GCGGG	0.521	0.062
GGTAC	0.521	0.075
GCGGA	0.521	0.049
AGCAG	0.520	0.025
TGCGA	0.520	0.023
AAAAT	0.520	0.028
AGCAA	0.519	0.070
AGAGA	0.519	0.029
AGTAG	0.518	0.062
GGAAT	0.518	0.045
ATGGG	0.517	0.030
AGCTA	0.517	0.044
TGCGT	0.516	0.061
CGGGT	0.516	0.027
GGATA	0.516	0.030
GCGGT	0.516	0.065
GGACG	0.516	0.039
AGCCA	0.515	0.031
TGAAA	0.514	0.049
GGCAT	0.514	0.040
AAGTC	0.514	0.030
TGGCT	0.513	0.034
AAATG	0.513	0.028

**AAGAG**	0.513	*0.026*
**GCGAA**	0.513	*0.033*
**GGAAG**	0.512	*0.026*
**AAGTG**	0.511	*0.032*
**GGTGT**	0.511	*0.029*
**GGACC**	0.511	*0.057*
**GGACA**	0.511	*0.053*
**CGGCC**	0.511	*0.041*
**TGTGG**	0.511	*0.037*
**GGATG**	0.510	*0.100*
**GGTAA**	0.510	*0.048*
**TGACG**	0.510	*0.055*
**GAGCG**	0.510	*0.085*
**AAGTA**	0.510	*0.053*
**GGCTC**	0.508	*0.037*
**GAGGA**	0.507	*0.034*
**GAGCA**	0.507	*0.078*
**AAATA**	0.507	*0.069*
**GGATC**	0.507	*0.036*
**TGTAA**	0.507	*0.031*
**GGCTG**	0.506	*0.027*
**TAGGG**	0.506	*0.024*
**CGGCG**	0.506	*0.029*
**GCCGC**	0.506	*0.046*
**ACGGG**	0.505	*0.024*
**GGCTA**	0.504	*0.027*
**AACGG**	0.504	*0.041*
**TGAGA**	0.503	*0.029*
**GGAAA**	0.502	*0.032*
**GCGAC**	0.502	*0.043*
**GAGCC**	0.501	*0.072*
**TGATT**	0.500	*0.030*
**AGTCC**	0.499	*0.086*
**GCGTG**	0.499	*0.028*
**AAACA**	0.499	*0.064*
**AAACG**	0.497	*0.030*
**TGTGT**	0.497	*0.026*
**TAGAA**	0.496	*0.081*
**GAGGT**	0.494	*0.035*
**GCGAT**	0.494	*0.055*

**TGTTG**	0.494	*0.027*
**ATGAA**	0.493	*0.074*
**AATGA**	0.493	*0.037*
**ACGGC**	0.493	*0.034*
**TGAAT**	0.493	*0.031*
**GAGAG**	0.492	*0.024*
**ATGGT**	0.492	*0.033*
**CGGAA**	0.492	*0.039*
**GCAGC**	0.492	*0.024*
**ATGAT**	0.491	*0.042*
**AGCAT**	0.491	*0.031*
**AAAAG**	0.491	*0.052*
**AGCTC**	0.490	*0.030*
**AAGCC**	0.490	*0.031*
**GCGTA**	0.490	*0.035*
**GGATT**	0.489	*0.055*
**AGATA**	0.489	*0.045*
**GGAAC**	0.489	*0.029*
**TGTAT**	0.489	*0.046*
**GAGAA**	0.489	*0.086*
**TTGGG**	0.489	*0.031*
**ATGGC**	0.488	*0.029*
**TGACT**	0.488	*0.050*
**TGCCG**	0.487	*0.022*
**GAGGC**	0.487	*0.024*
**AAATT**	0.487	*0.042*
**ATGTT**	0.487	*0.053*
**CGGGC**	0.487	*0.024*
**ATGAG**	0.486	*0.035*
**TGTTA**	0.486	*0.037*
**GGACT**	0.486	*0.034*
**GGCTT**	0.485	*0.024*
**TCGCG**	0.485	*0.025*
**GTGGC**	0.484	*0.031*
**AAAGA**	0.484	*0.055*
**TGCCT**	0.484	*0.027*
**GGTGC**	0.483	*0.032*
**GGTGG**	0.483	*0.055*
**ACGTG**	0.483	*0.028*
**GCGTC**	0.483	*0.051*

**GGTAG**	0.482	*0.023*
**TGCAT**	0.482	*0.038*
**GTGGA**	0.482	*0.017*
**TGATA**	0.482	*0.041*
**GGTCA**	0.481	*0.023*
**AAGAA**	0.481	*0.022*
**ATAGT**	0.481	*0.032*
**TGCCC**	0.480	*0.021*
**CGCGT**	0.480	*0.034*
**ATAGG**	0.480	*0.045*
**GAGTC**	0.479	*0.041*
**TGCCA**	0.479	*0.034*
**TGTAG**	0.478	*0.040*
**CGGGA**	0.478	*0.064*
**GAGCT**	0.478	*0.035*
**GAGGG**	0.478	*0.026*
**AATGT**	0.478	*0.030*
**TGAAC**	0.478	*0.039*
**TTGGT**	0.477	*0.037*
**GAGAT**	0.477	*0.059*
**TGAGC**	0.477	*0.058*
**AAAAA**	0.476	*0.026*
**GGTTA**	0.476	*0.055*
**ACGCC**	0.475	*0.024*
**AAAGC**	0.475	*0.057*
**AGTCA**	0.475	*0.024*
**GCATG**	0.475	*0.031*
**TAGCG**	0.474	*0.037*
**TGCAG**	0.474	*0.028*
**TGACA**	0.474	*0.030*
**TGTGA**	0.474	*0.033*
**TGCAC**	0.473	*0.030*
**GAGTG**	0.472	*0.057*
**ACGGT**	0.472	*0.032*
**GTGCA**	0.472	*0.028*
**TAGGC**	0.472	*0.033*
**AGCAC**	0.471	*0.023*
**GGTTG**	0.471	*0.028*
**AAGAC**	0.471	*0.027*
**TAGTA**	0.471	*0.030*

**CGGCT**	0.470	*0.042*
**GGTCC**	0.470	*0.054*
**GGTTC**	0.470	*0.050*
**ATGTA**	0.470	*0.039*
**GGTCG**	0.470	*0.032*
**AAAAC**	0.470	*0.030*
**CGGAC**	0.469	*0.032*
**TAGAC**	0.469	*0.021*
**GGTAT**	0.466	*0.023*
**ATGCG**	0.466	*0.026*
**GAGTA**	0.466	*0.040*
**TGTGC**	0.466	*0.037*
**ATGCA**	0.465	*0.024*
**ATGGA**	0.465	*0.060*
**CGGAG**	0.465	*0.033*
**CGGAT**	0.464	*0.026*
**TGTAC**	0.464	*0.031*
**TTGGA**	0.464	*0.080*
**TAGAT**	0.463	*0.020*
**AACGT**	0.463	*0.026*
**GCTGC**	0.463	*0.024*
**GAAGC**	0.463	*0.021*
**AATCG**	0.463	*0.032*
**ACGGA**	0.463	*0.017*
**TAGGA**	0.463	*0.028*
**GTGAC**	0.463	*0.025*
**AAACT**	0.462	*0.051*
**CGCAG**	0.462	*0.065*
**GAGAC**	0.462	*0.030*
**TAAAA**	0.462	*0.077*
**GAATG**	0.462	*0.069*
**ATGAC**	0.461	*0.022*
**ACGTA**	0.460	*0.036*
**GCGTT**	0.460	*0.036*
**TGTTT**	0.460	*0.028*
**GATGA**	0.460	*0.032*
**AGACA**	0.459	*0.036*
**TGATG**	0.459	*0.032*
**TGTCC**	0.459	*0.038*
**TGACC**	0.458	*0.021*

**AATGG**	0.458	*0.031*
**TAGGT**	0.458	*0.036*
**TGTCG**	0.458	*0.039*
**ATAAT**	0.458	*0.047*
**ATGCT**	0.457	*0.043*
**AAATC**	0.457	*0.021*
**CGGTT**	0.457	*0.023*
**GTGAA**	0.457	*0.041*
**GAAGA**	0.456	*0.024*
**ACGTT**	0.456	*0.039*
**GTGGT**	0.456	*0.022*
**TAGTC**	0.455	*0.036*
**GCAGG**	0.455	*0.019*
**GTGAT**	0.455	*0.053*
**TAAAT**	0.454	*0.038*
**AATCT**	0.454	*0.047*
**AACTG**	0.453	*0.040*
**TAGTT**	0.453	*0.030*
**TAAGG**	0.453	*0.043*
**GTGTA**	0.452	*0.034*
**AAGCA**	0.452	*0.026*
**AATAG**	0.452	*0.031*
**ACGTC**	0.452	*0.046*
**ACGAA**	0.451	*0.026*
**TGCTG**	0.451	*0.032*
**ATAAA**	0.451	*0.031*
**TGATC**	0.451	*0.029*
**TGCAA**	0.450	*0.024*
**TGTCA**	0.450	*0.060*
**GCCGG**	0.449	*0.043*
**TGCTA**	0.449	*0.027*
**TAAGA**	0.449	*0.024*
**ACGAC**	0.448	*0.027*
**ACGAT**	0.447	*0.024*
**ATTGG**	0.446	*0.071*
**AATAA**	0.446	*0.022*
**GACGC**	0.446	*0.029*
**AATTG**	0.446	*0.027*
**TTGCC**	0.446	*0.059*
**GAAGG**	0.445	*0.030*

**AAACC**	0.445	*0.050*
**GTGAG**	0.445	*0.054*
**CGGTA**	0.445	*0.026*
**ACGCA**	0.445	*0.028*
**GGTTT**	0.444	*0.027*
**GCTGA**	0.444	*0.036*
**CGCTG**	0.444	*0.032*
**GATGT**	0.443	*0.063*
**GCAGA**	0.443	*0.033*
**AACGC**	0.443	*0.035*
**TCGGT**	0.442	*0.053*
**TCGGG**	0.442	*0.024*
**AATAT**	0.441	*0.069*
**CGATG**	0.441	*0.081*
**GTGCT**	0.441	*0.029*
**TAGCT**	0.441	*0.022*
**GAAGT**	0.440	*0.024*
**CGAGG**	0.439	*0.028*
**CGCAT**	0.439	*0.032*
**AATTT**	0.439	*0.024*
**CGCTA**	0.439	*0.020*
**CGGTG**	0.439	*0.041*
**TAGAG**	0.439	*0.023*
**GTGCG**	0.439	*0.023*
**GCCAT**	0.439	*0.027*
**ACTGT**	0.439	*0.080*
**GTAGA**	0.439	*0.018*
**CGGTC**	0.439	*0.035*
**GATTG**	0.438	*0.067*
**CGCAC**	0.438	*0.036*
**GTAGC**	0.438	*0.023*
**GCCGA**	0.437	*0.029*
**TAAGT**	0.436	*0.035*
**CGCAA**	0.436	*0.057*
**ATGCC**	0.436	*0.028*
**TTGTG**	0.435	*0.082*
**TCGGA**	0.435	*0.028*
**CGAGA**	0.435	*0.046*
**GATGC**	0.435	*0.030*
**TAATG**	0.435	*0.043*

**ACAGG**	0.435	*0.053*
**GTGCC**	0.435	*0.029*
**TAGCC**	0.435	*0.022*
**TTGAC**	0.434	*0.015*
**ATGTG**	0.434	*0.027*
**ATAGA**	0.434	*0.019*
**CGCCG**	0.434	*0.033*
**ACGCT**	0.434	*0.029*
**AACAT**	0.433	*0.059*
**GATCA**	0.433	*0.025*
**TTGAA**	0.433	*0.024*
**TTGCA**	0.433	*0.029*
**GCAGT**	0.433	*0.063*
**CGGCA**	0.433	*0.049*
**TGTCT**	0.433	*0.020*
**ATTTA**	0.432	*0.031*
**TTGTA**	0.432	*0.035*
**TGCTT**	0.432	*0.025*
**AATCC**	0.432	*0.031*
**GATGG**	0.431	*0.046*
**ATACG**	0.431	*0.024*
**TCGGC**	0.431	*0.021*
**ATATA**	0.431	*0.042*
**GAAAG**	0.431	*0.056*
**GTGTG**	0.430	*0.030*
**ATTAA**	0.430	*0.027*
**GGTCT**	0.430	*0.035*
**GCATA**	0.430	*0.029*
**ACGAG**	0.430	*0.036*
**ACTAG**	0.430	*0.022*
**TAGCA**	0.429	*0.045*
**GCCTG**	0.429	*0.081*
**TAGTG**	0.429	*0.021*
**GAGTT**	0.429	*0.016*
**ACAGC**	0.429	*0.053*
**GAATA**	0.429	*0.031*
**TGTTC**	0.429	*0.024*
**ATGTC**	0.429	*0.072*
**GTGGG**	0.428	*0.024*
**GCCAG**	0.428	*0.043*

**AATAC**	0.428	*0.023*
**AATGC**	0.428	*0.024*
**TCGCC**	0.428	*0.034*
**AACCG**	0.427	*0.041*
**AACGA**	0.427	*0.030*
**GCCTA**	0.427	*0.035*
**ATAGC**	0.427	*0.025*
**GAATC**	0.427	*0.031*
**AATTC**	0.427	*0.039*
**TTGGC**	0.427	*0.024*
**GCCTC**	0.426	*0.028*
**CGAAC**	0.426	*0.070*
**GCAAA**	0.426	*0.028*
**GCAAT**	0.426	*0.037*
**CGAAA**	0.426	*0.021*
**GACGG**	0.426	*0.017*
**CGAGC**	0.425	*0.035*
**GAACA**	0.425	*0.021*
**GATAG**	0.425	*0.049*
**ACCGG**	0.425	*0.025*
**GTGTC**	0.425	*0.020*
**CGTAG**	0.425	*0.030*
**GACGT**	0.425	*0.029*
**GTCGA**	0.424	*0.058*
**ATTAG**	0.424	*0.034*
**GACAG**	0.423	*0.036*
**GCCAC**	0.423	*0.058*
**ACCGC**	0.423	*0.051*
**GTCGC**	0.423	*0.043*
**TTGCG**	0.422	*0.024*
**GATAC**	0.422	*0.027*
**GAACG**	0.422	*0.028*
**GTTGC**	0.422	*0.051*
**GCACA**	0.422	*0.054*
**AACTT**	0.422	*0.055*
**CGAGT**	0.422	*0.080*
**GATCG**	0.422	*0.053*
**TTGAG**	0.422	*0.024*
**ATAAC**	0.422	*0.042*
**GATTA**	0.421	*0.058*

**GACAA**	0.421	*0.019*
**GCCAA**	0.420	*0.035*
**TCGAT**	0.420	*0.035*
**GACGA**	0.420	*0.020*
**ATCGG**	0.420	*0.028*
**ATAAG**	0.420	*0.053*
**TAAAG**	0.419	*0.025*
**GTATG**	0.419	*0.027*
**TTCGA**	0.419	*0.041*
**AACAA**	0.419	*0.024*
**CGCCC**	0.419	*0.034*
**CGTGG**	0.419	*0.027*
**TTAGT**	0.419	*0.050*
**GCTCA**	0.418	*0.030*
**GATAT**	0.418	*0.032*
**TATTA**	0.418	*0.024*
**TATGT**	0.418	*0.031*
**TCGAC**	0.418	*0.022*
**TAATA**	0.418	*0.026*
**CGAAT**	0.417	*0.029*
**TTGCT**	0.417	*0.026*
**TATGA**	0.417	*0.028*
**GCATC**	0.416	*0.022*
**AATTA**	0.416	*0.035*
**TGCTC**	0.416	*0.024*
**ATCAG**	0.416	*0.029*
**ATTGT**	0.415	*0.023*
**AACAG**	0.415	*0.022*
**TCGTG**	0.414	*0.050*
**ACTGG**	0.414	*0.020*
**GATCC**	0.414	*0.023*
**AATCA**	0.413	*0.021*
**GACAT**	0.413	*0.030*
**TATAA**	0.413	*0.036*
**CGCCT**	0.412	*0.025*
**TCAGG**	0.412	*0.040*
**CGCTC**	0.412	*0.034*
**TAATT**	0.412	*0.019*
**GAATT**	0.411	*0.026*
**TTGTC**	0.411	*0.033*
**AACTA**	0.411	*0.035*
**GACTA**	0.411	*0.030*
**GCCCA**	0.411	*0.030*
**AACAC**	0.411	*0.021*
**ATTTT**	0.411	*0.028*
**TTGAT**	0.411	*0.032*
**ATATC**	0.411	*0.023*
**TATGG**	0.411	*0.025*
**ACAGT**	0.411	*0.026*
**TTAGA**	0.410	*0.018*
**TCGAG**	0.410	*0.022*
**GCTAG**	0.410	*0.025*
**TAAAC**	0.410	*0.061*
**TTAGG**	0.410	*0.061*
**TTCGC**	0.409	*0.047*
**ATTAT**	0.409	*0.035*
**GTACA**	0.409	*0.023*
**ATTGA**	0.409	*0.016*
**GTAGT**	0.409	*0.037*
**GTTGA**	0.408	*0.034*
**ATTCG**	0.408	*0.030*
**GATAA**	0.408	*0.017*
**GCAAC**	0.408	*0.039*
**TACTA**	0.408	*0.028*
**CTGGC**	0.407	*0.042*
**GTATA**	0.407	*0.024*
**ACTAT**	0.407	*0.053*
**ACAAA**	0.407	*0.023*
**ACTGA**	0.407	*0.029*
**CGATA**	0.407	*0.031*
**CGATC**	0.407	*0.040*
**CGCTT**	0.406	*0.088*
**CGCCA**	0.406	*0.026*
**TACGG**	0.406	*0.025*
**TAACG**	0.406	*0.027*
**ATCAA**	0.406	*0.019*
**ACAAT**	0.405	*0.033*
**TTGTT**	0.405	*0.024*
**CGTGA**	0.405	*0.024*
**TATAT**	0.405	*0.037*
**TACGA**	0.405	*0.032*
**TTCGG**	0.405	*0.023*
**GCTGG**	0.405	*0.028*
**ATATG**	0.404	*0.027*
**GCAAG**	0.404	*0.023*
**CGACA**	0.404	*0.024*
**ATCTT**	0.404	*0.036*
**TACCG**	0.404	*0.029*
**TTTGA**	0.404	*0.022*
**TTAGC**	0.404	*0.025*
**CAGGT**	0.404	*0.046*
**GACTG**	0.404	*0.051*
**TTATT**	0.403	*0.029*
**CGTCG**	0.403	*0.029*
**ATTTG**	0.403	*0.022*
**ACCGT**	0.403	*0.029*
**GAAAA**	0.403	*0.030*
**ATACA**	0.403	*0.038*
**TCGAA**	0.402	*0.026*
**CGACG**	0.402	*0.045*
**CGTGT**	0.402	*0.027*
**TAAGC**	0.402	*0.026*
**TTTGG**	0.402	*0.045*
**CGTTA**	0.402	*0.051*
**TTATC**	0.402	*0.056*
**AACCA**	0.402	*0.022*
**GCTAT**	0.402	*0.031*
**CAGGG**	0.401	*0.025*
**GATCT**	0.401	*0.031*
**TTCAG**	0.401	*0.021*
**TATGC**	0.401	*0.064*
**TTAAA**	0.401	*0.019*
**ATTTC**	0.400	*0.037*
**ATCGA**	0.400	*0.019*
**GTTCA**	0.400	*0.024*
**ATATT**	0.400	*0.028*
**CGTAA**	0.400	*0.035*
**CCGCG**	0.400	*0.041*
**ATCGT**	0.399	*0.041*
**GCACT**	0.399	*0.022*
**ATCGC**	0.399	*0.027*
**TCATG**	0.399	*0.025*
**GCTAA**	0.399	*0.026*
**ACATG**	0.399	*0.019*
**GTCAC**	0.398	*0.035*
**ATTAC**	0.398	*0.027*
**GCATT**	0.398	*0.080*
**ATTGC**	0.398	*0.052*
**GTGTT**	0.397	*0.019*
**CGAAG**	0.397	*0.027*
**CGTGC**	0.397	*0.027*
**GAAAT**	0.397	*0.021*
**TATAC**	0.397	*0.025*
**ACATT**	0.397	*0.025*
**GAAAC**	0.396	*0.026*
**TTTGT**	0.396	*0.060*
**TAACA**	0.396	*0.021*
**ATACT**	0.396	*0.024*
**TTACG**	0.395	*0.019*
**CGACC**	0.395	*0.020*
**AACTC**	0.395	*0.020*
**GATTC**	0.395	*0.039*
**TACGC**	0.395	*0.021*
**CGTAC**	0.395	*0.026*
**CAGCG**	0.394	*0.023*
**CGTCC**	0.394	*0.032*
**ACCGA**	0.394	*0.023*
**TAATC**	0.394	*0.027*
**TAACC**	0.394	*0.022*
**ACAAG**	0.394	*0.024*
**GCCCG**	0.394	*0.045*
**TACGT**	0.394	*0.020*
**ACCAG**	0.394	*0.024*
**GATTT**	0.394	*0.048*
**TCCGA**	0.394	*0.083*
**CCGCA**	0.393	*0.027*
**TTTGC**	0.393	*0.026*
**TCGTA**	0.393	*0.020*
**GCTCC**	0.393	*0.056*
**CGACT**	0.393	*0.047*

**GTTGG**	0.392	*0.022*	**GCTTC**	0.384	*0.019*	**GCTCT**	0.376	*0.039*	**CGTCT**	0.368	*0.024*
**GCTGT**	0.392	*0.023*	**TTACA**	0.384	*0.021*	**CAGTA**	0.376	*0.020*	**TACAC**	0.368	*0.016*
**TATCA**	0.392	*0.021*	**GTCCA**	0.384	*0.020*	**TATCG**	0.376	*0.035*	**TTTTG**	0.368	*0.022*
**TCGCT**	0.392	*0.085*	**TTTCG**	0.384	*0.038*	**CATGC**	0.376	*0.053*	**TCTGA**	0.368	*0.029*
**TTATA**	0.392	*0.024*	**CAGGA**	0.383	*0.033*	**ACACG**	0.375	*0.026*	**GTAAG**	0.367	*0.029*
**TCGCA**	0.391	*0.029*	**ACAGA**	0.383	*0.029*	**GTAAA**	0.375	*0.035*	**TCTTG**	0.367	*0.027*
**TCTGG**	0.391	*0.031*	**GTATC**	0.383	*0.029*	**TTATG**	0.375	*0.030*	**CCGGG**	0.367	*0.032*
**TCGTC**	0.390	*0.049*	**TTTAG**	0.383	*0.028*	**ACACC**	0.375	*0.055*	**TTCCG**	0.367	*0.035*
**GAACT**	0.390	*0.042*	**GCACG**	0.383	*0.036*	**ACCAA**	0.375	*0.053*	**TCTGT**	0.367	*0.028*
**TACTG**	0.390	*0.030*	**ACTTC**	0.382	*0.041*	**ACCAC**	0.375	*0.043*	**ATCCG**	0.366	*0.039*
**GTCAG**	0.390	*0.032*	**ACTAA**	0.382	*0.028*	**GCTAC**	0.375	*0.026*	**CTGCG**	0.366	*0.028*
**TTTAA**	0.390	*0.025*	**TTAAG**	0.382	*0.030*	**GCTCG**	0.375	*0.032*	**GTCCT**	0.366	*0.034*
**GCCGT**	0.389	*0.029*	**TATAG**	0.382	*0.018*	**ACACA**	0.374	*0.031*	**CAAGC**	0.366	*0.022*
**ACTTG**	0.389	*0.044*	**TACAT**	0.382	*0.025*	**GACCC**	0.374	*0.025*	**ATCCA**	0.366	*0.019*
**ACCCG**	0.389	*0.058*	**GCCCT**	0.381	*0.040*	**CAGCC**	0.374	*0.068*	**ACCTG**	0.366	*0.023*
**CGTTG**	0.389	*0.025*	**GTCAA**	0.381	*0.029*	**TACAA**	0.374	*0.021*	**GCCTT**	0.366	*0.035*
**TCAGA**	0.389	*0.039*	**TTTTA**	0.381	*0.020*	**GTTGT**	0.373	*0.017*	**GTATT**	0.366	*0.024*
**TATTG**	0.389	*0.017*	**GTTAA**	0.381	*0.025*	**GTCAT**	0.373	*0.027*	**CGTTC**	0.365	*0.022*
**CAGGC**	0.389	*0.034*	**ATCTG**	0.381	*0.022*	**ACCTA**	0.373	*0.038*	**CGTCA**	0.365	*0.028*
**GACTC**	0.389	*0.030*	**TTTAT**	0.381	*0.021*	**ACTTT**	0.373	*0.046*	**TATTT**	0.365	*0.017*
**AACCT**	0.388	*0.025*	**ATACC**	0.380	*0.033*	**ATTCT**	0.373	*0.022*	**CCGGC**	0.364	*0.027*
**TACAG**	0.388	*0.025*	**TTTAC**	0.380	*0.031*	**CTGAC**	0.373	*0.086*	**GTCGG**	0.364	*0.018*
**GCTTA**	0.388	*0.021*	**CAGCA**	0.380	*0.035*	**CTGCA**	0.373	*0.025*	**GTAAC**	0.364	*0.026*
**ATCAT**	0.388	*0.028*	**ACACT**	0.379	*0.034*	**ACATA**	0.372	*0.024*	**GTCCC**	0.364	*0.024*
**GTAGG**	0.388	*0.026*	**ATTCA**	0.379	*0.027*	**ACTAC**	0.372	*0.026*	**CAATG**	0.364	*0.026*
**GTCTG**	0.387	*0.025*	**ACTGC**	0.378	*0.032*	**CAGAC**	0.372	*0.017*	**ACATC**	0.364	*0.024*
**CAGTG**	0.387	*0.064*	**TCATA**	0.378	*0.019*	**GCTTG**	0.371	*0.032*	**ACCAT**	0.364	*0.020*
**CTGGT**	0.387	*0.040*	**TACTC**	0.378	*0.019*	**GTCTA**	0.371	*0.036*	**TACCA**	0.364	*0.024*
**GCACC**	0.386	*0.021*	**GTTCC**	0.378	*0.025*	**CATGA**	0.371	*0.027*	**TTCTG**	0.364	*0.030*
**ACTCG**	0.386	*0.032*	**TACTT**	0.378	*0.021*	**GTTCT**	0.371	*0.032*	**GTAAT**	0.363	*0.023*
**AACCC**	0.386	*0.022*	**GACCA**	0.378	*0.028*	**TATCT**	0.370	*0.029*	**GTTAC**	0.363	*0.021*
**TAACT**	0.386	*0.019*	**CAGCT**	0.378	*0.038*	**TTTCA**	0.370	*0.022*	**CAGAA**	0.363	*0.018*
**TCCGG**	0.386	*0.035*	**TTAAT**	0.377	*0.027*	**CCGGT**	0.369	*0.031*	**CTGAT**	0.363	*0.023*
**CGATT**	0.385	*0.023*	**GCCCC**	0.377	*0.026*	**TCCGC**	0.369	*0.030*	**TCAGC**	0.363	*0.028*
**GTACG**	0.385	*0.023*	**GTACT**	0.377	*0.019*	**CAGAT**	0.369	*0.015*	**TTCAA**	0.362	*0.026*
**TTAAC**	0.385	*0.029*	**GACCT**	0.377	*0.020*	**ATTCC**	0.369	*0.029*	**TATCC**	0.361	*0.028*
**GACAC**	0.384	*0.019*	**CTGGG**	0.377	*0.024*	**TATTC**	0.369	*0.025*	**TCGTT**	0.361	*0.019*
**TTCGT**	0.384	*0.038*	**CGTAT**	0.377	*0.024*	**ACAAC**	0.368	*0.022*	**GTACC**	0.361	*0.035*
**GAACC**	0.384	*0.029*	**ACTCC**	0.377	*0.042*	**CCGGA**	0.368	*0.064*	**TCATT**	0.361	*0.047*
**GACCG**	0.384	*0.031*	**CTGGA**	0.376	*0.019*	**ATCTA**	0.368	*0.025*	**TCCAA**	0.360	*0.039*

**TCTGC**	0.360	*0.015*
**ATCTC**	0.360	*0.023*
**TCAAG**	0.360	*0.026*
**GTTAT**	0.359	*0.021*
**TTACT**	0.359	*0.028*
**TTCCC**	0.359	*0.025*
**CTGAG**	0.359	*0.017*
**CTAGA**	0.359	*0.023*
**CAAGA**	0.358	*0.022*
**TTCAT**	0.358	*0.039*
**GTCGT**	0.358	*0.022*
**GACTT**	0.358	*0.027*
**TCAGT**	0.357	*0.022*
**TTTTC**	0.357	*0.035*
**TTACC**	0.357	*0.020*
**CAGAG**	0.357	*0.018*
**TACCC**	0.356	*0.015*
**ACCTC**	0.356	*0.055*
**CTGTG**	0.356	*0.068*
**TCCGT**	0.356	*0.021*
**GTTAG**	0.355	*0.024*
**ATCCT**	0.355	*0.019*
**GTTTA**	0.355	*0.025*
**TCTAG**	0.354	*0.017*
**CTAGC**	0.354	*0.027*
**CAGTC**	0.354	*0.033*
**CAGTT**	0.354	*0.040*
**TTTCT**	0.354	*0.032*
**TCAAT**	0.353	*0.027*
**GTCTC**	0.352	*0.033*
**CAAGG**	0.352	*0.019*
**CTGTC**	0.352	*0.018*
**ATCAC**	0.352	*0.025*
**CTGCT**	0.352	*0.030*
**ACCCC**	0.352	*0.021*
**TTCAC**	0.351	*0.022*
**TTCCT**	0.351	*0.029*
**TTCCA**	0.351	*0.018*
**CTGTA**	0.351	*0.046*
**ACCCA**	0.351	*0.020*

**ACTTA**	0.351	*0.021*
**TTCTA**	0.350	*0.024*
**ACCTT**	0.350	*0.028*
**CTAGG**	0.350	*0.055*
**ATCCC**	0.350	*0.019*
**GTCCG**	0.350	*0.020*
**TCACA**	0.350	*0.033*
**TCAAA**	0.349	*0.028*
**CATGG**	0.349	*0.037*
**CTGAA**	0.349	*0.015*
**ACTCT**	0.348	*0.022*
**TCCTA**	0.348	*0.020*
**TTCTT**	0.348	*0.026*
**TTTCC**	0.347	*0.023*
**TCCTG**	0.346	*0.024*
**ACTCA**	0.346	*0.028*
**ACCCT**	0.346	*0.035*
**TCCAT**	0.345	*0.023*
**CTGTT**	0.345	*0.024*
**CAAGT**	0.345	*0.021*
**CTGCC**	0.345	*0.018*
**CATGT**	0.344	*0.022*
**TACCT**	0.344	*0.024*
**GTTTC**	0.343	*0.027*
**GTTCG**	0.343	*0.021*
**CACGC**	0.342	*0.060*
**TCTTC**	0.342	*0.027*
**TCAAC**	0.342	*0.018*
**CGTTT**	0.341	*0.032*
**TTTTT**	0.340	*0.015*
**TCCCA**	0.340	*0.021*
**TCCAG**	0.340	*0.026*
**TCACG**	0.339	*0.024*
**TCTAA**	0.338	*0.020*
**CATTG**	0.338	*0.055*
**GTCTT**	0.338	*0.046*
**CATCG**	0.338	*0.031*
**CTCGG**	0.337	*0.050*
**TCTCG**	0.337	*0.023*
**CCGCT**	0.337	*0.022*

**CAAAT**	0.336	*0.028*
**CACGG**	0.336	*0.030*
**CACTG**	0.336	*0.022*
**CTTGA**	0.335	*0.025*
**CTAGT**	0.335	*0.028*
**CATTA**	0.334	*0.032*
**CATAG**	0.334	*0.024*
**TCACT**	0.334	*0.020*
**GTTTT**	0.334	*0.032*
**TTCTC**	0.334	*0.024*
**CAAAG**	0.333	*0.045*
**CATAT**	0.333	*0.035*
**CCGTG**	0.332	*0.028*
**TCTCT**	0.332	*0.024*
**CCAGA**	0.331	*0.020*
**TCTCA**	0.331	*0.022*
**TCCTC**	0.330	*0.032*
**GTTTG**	0.330	*0.029*
**TCTAT**	0.330	*0.017*
**TCATC**	0.330	*0.024*
**CTCTG**	0.330	*0.043*
**CCGCC**	0.330	*0.022*
**TCTAC**	0.330	*0.034*
**TCCCG**	0.329	*0.019*
**CCTGA**	0.329	*0.040*
**CATCA**	0.328	*0.035*
**CACGA**	0.328	*0.012*
**GCTTT**	0.327	*0.027*
**CAATA**	0.327	*0.023*
**TCCAC**	0.326	*0.030*
**TCTTA**	0.326	*0.021*
**CACTA**	0.325	*0.032*
**TCCTT**	0.325	*0.036*
**CCAGC**	0.324	*0.030*
**CAAAC**	0.323	*0.031*
**CTCGA**	0.323	*0.024*
**TCACC**	0.322	*0.023*
**CCGTT**	0.322	*0.059*
**CTATA**	0.322	*0.017*
**CTACA**	0.322	*0.018*

**CACAG**	0.321	*0.044*
**CTTGC**	0.321	*0.028*
**CAAAA**	0.321	*0.019*
**CACGT**	0.321	*0.028*
**TCTTT**	0.321	*0.019*
**CCGAC**	0.321	*0.042*
**CCGTA**	0.319	*0.020*
**CAACG**	0.319	*0.017*
**CAACT**	0.318	*0.025*
**CTCGC**	0.318	*0.024*
**CCTGG**	0.317	*0.030*
**CCAGG**	0.317	*0.023*
**CTCAG**	0.317	*0.028*
**CATAA**	0.317	*0.019*
**CTTAG**	0.317	*0.020*
**TCCCT**	0.317	*0.026*
**CAATC**	0.316	*0.032*
**CCTGC**	0.315	*0.021*
**CACTC**	0.314	*0.022*
**CAATT**	0.314	*0.026*
**CATAC**	0.314	*0.032*
**CCGAA**	0.312	*0.023*
**TCCCC**	0.312	*0.016*
**CCCGG**	0.312	*0.028*
**CCGTC**	0.312	*0.018*
**CTTCA**	0.312	*0.027*
**CACAT**	0.312	*0.014*
**TCTCC**	0.312	*0.026*
**CCGAG**	0.312	*0.029*
**CTATT**	0.312	*0.018*
**CACAC**	0.311	*0.024*
**CCATG**	0.311	*0.017*
**CTAAG**	0.311	*0.025*
**CCGAT**	0.311	*0.015*
**CTTGG**	0.310	*0.025*
**CTTGT**	0.310	*0.025*
**CTATG**	0.309	*0.019*
**CTATC**	0.309	*0.018*
**CCCGC**	0.308	*0.055*
**CTTTA**	0.307	*0.019*

**CACAA**	0.306	*0.021*
**CTAAA**	0.306	*0.019*
**CATCC**	0.306	*0.042*
**CTTTG**	0.306	*0.036*
**CTACT**	0.305	*0.028*
**CCCGA**	0.304	*0.018*
**CTAAT**	0.303	*0.015*
**CAACA**	0.302	*0.032*
**CATTC**	0.300	*0.017*
**CCAGT**	0.300	*0.020*
**CCTCA**	0.300	*0.044*
**CACCG**	0.299	*0.015*
**CTTAC**	0.297	*0.021*
**CTTAA**	0.297	*0.018*
**CTTTC**	0.297	*0.050*
**CATCT**	0.296	*0.017*
**CTCAC**	0.296	*0.022*
**CTCGT**	0.296	*0.036*
**CCATA**	0.295	*0.018*
**CCCTG**	0.294	*0.019*

**CCCAA**	0.294	*0.016*
**CACCC**	0.294	*0.044*
**CCTAG**	0.294	*0.027*
**CTCAA**	0.293	*0.019*
**CTTAT**	0.292	*0.018*
**CATTT**	0.292	*0.021*
**CTCTA**	0.291	*0.020*
**CAACC**	0.291	*0.020*
**CCTAA**	0.290	*0.018*
**CCAAA**	0.290	*0.015*
**CACTT**	0.289	*0.016*
**CCTGT**	0.289	*0.018*
**CTACG**	0.288	*0.026*
**CCAAG**	0.288	*0.027*
**CTTCC**	0.288	*0.030*
**CCATT**	0.286	*0.017*
**CACCA**	0.286	*0.018*
**CTCCG**	0.286	*0.060*
**CCTCG**	0.286	*0.021*
**CTTCG**	0.285	*0.024*

**CCTAT**	0.285	*0.019*
**CTCTC**	0.284	*0.019*
**CTAAC**	0.284	*0.012*
**CCATC**	0.284	*0.016*
**CCCAG**	0.282	*0.022*
**CTTCT**	0.282	*0.018*
**CTACC**	0.281	*0.020*
**CCACG**	0.280	*0.022*
**CCAAT**	0.278	*0.021*
**CCCAT**	0.276	*0.019*
**CCTTG**	0.275	*0.015*
**CCACA**	0.275	*0.012*
**CCACC**	0.275	*0.026*
**CCTAC**	0.273	*0.017*
**CACCT**	0.273	*0.016*
**CCTCC**	0.273	*0.021*
**CTCAT**	0.273	*0.018*
**CCCGT**	0.272	*0.016*
**CTCTT**	0.272	*0.035*
**CCCCG**	0.272	*0.012*

**CCTTA**	0.272	*0.020*
**CTCCA**	0.271	*0.016*
**CCCAC**	0.271	*0.019*
**CCCCA**	0.271	*0.027*
**CCCTC**	0.269	*0.018*
**CCTCT**	0.268	*0.019*
**CCCTT**	0.266	*0.016*
**CTCCT**	0.266	*0.019*
**CCACT**	0.265	*0.016*
**CCAAC**	0.265	*0.023*
**CTCCC**	0.260	*0.019*
**CTTTT**	0.257	*0.015*
**CCTTT**	0.253	*0.015*
**CCCCC**	0.251	*0.018*
**CCCTA**	0.247	*0.016*
**CCCCT**	0.243	*0.015*
**CCTTC**	0.229	*0.015*

References

1. C. Agbavwe, C. Kim, D. Hong, K. Heinrich, T. Wang and M. M. Somoza, *J. Nanobiotechnol.*, 2011, **9**.
2. M. Sack, N. Kretschy, B. Rohm, V. Somoza and M. M. Somoza, *Anal. Chem.*, 2013, **85**, 8513-8517.
3. N. Kretschy, A. K. Holik, V. Somoza, K. P. Stengele and M. M. Somoza, *Angew. Chem. Int. Ed.*, 2015, **54**, 8555-8559.
4. M. Sack, K. Holz, A. K. Holik, N. Kretschy, V. Somoza, K. P. Stengele and M. M. Somoza, *J. Nanobiotechnol.*, 2016, **14**, 1-13.
5. K. Holz, J. Lietard and M. M. Somoza, *ACS Sustain. Chem. Eng.*, 2017, **5**, 828-834.
6. J. Lietard, M. J. Damha and M. M. Somoza, in *Enzymatic and Chemical Synthesis of Nucleic Acid Derivatives*, ed. J. Fernández-Lucas, 2018, DOI: 10.1002/9783527812103.ch11.
7. J. Lietard, A. Leger, Y. Erlich, N. Sadowski, W. Timp and M. M. Somoza, *Nucleic Acids Res.*, 2021, **49**, 6687-6701.
8. K. Holz, J. K. Hoi, E. Schaudy, V. Somoza, J. Lietard and M. M. Somoza, *Sci. Rep.*, 2018, **8**, 15099.
